# Risk factors of periodontal disease in maintenance hemodialysis patients

**DOI:** 10.1097/MD.0000000000007892

**Published:** 2017-09-01

**Authors:** Yue Hou, Xin Wang, Cong-Xiao Zhang, Yu-Dan Wei, Li-Li Jiang, Xiao-Yu Zhu, Yu-Jun Du

**Affiliations:** a Department of Nephrology, First Hospital of Jilin University, Changchun, Jilin; b Department of Nephrology, Panjin Center Hospital, Panjin, Liaoning; c Department of Stomatology, First Hospital of Jilin University, Changchun, Jilin, China.

**Keywords:** aging, hemodialysis, periodontal disease, risk factor

## Abstract

To explore the characteristics and relevant risk factors of periodontal disease (PD) among hemodialysis patients.

Uremic patients on maintenance hemodialysis from November 2015 to March 2016 were retrospectively reviewed. Patients were divided into a PD group and a non-PD group. Demographic and laboratory data were collected and analyzed.

In all, 136 uremic patients (79 males and 57 females, aged 50.8 ± 15.3 years) on maintenance hemodialysis were included in this study. The incidence of PD increased with age. Hemodialysis patients most likely developed PD if they were male, smokers, or diabetic (*P* = .009, <.001, and <.001, respectively). Patients brushing their teeth twice daily had significantly less chance of developing PD as compared with those only brushing once daily (*P* < .001). Hemodialysis patients in the PD group had significantly higher levels of total cholesterol, high-sensitivity C-reactive protein, fasting blood glucose, and peripheral white blood cell counts, compared with the non-PD group (all *P* < .001). Logistic regression analysis revealed that diabetes, total cholesterol, high-sensitivity C-reactive protein, and peripheral white blood cell count were independent risk factors for developing PD, whereas teeth brushing twice daily and serum calcium were favorable factors for maintenance hemodialysis patients against PD.

Identification of risk factors provides a theoretical basis for prevention and improvement of PD among maintenance hemodialysis patients.

## 1 Introduction

End-stage renal disease (ESRD) is the final stage of chronic kidney disease, characterized by severe renal impairment, abnormal biochemical indicators, and endocrinal and metabolic disorders.^[1]^ A large number of studies have shown a close and complex link between periodontal disease (PD) and chronic kidney disease.^[2–4]^ Patients with severe PD exhibit elevated serum creatinine and a two-fold increased risk of the occurrence of chronic renal insufficiency as compared with non-PD patients.^[5]^ Once oral pathogens and toxins enter the bloodstream through the periodontal pocket, they cause a pathologic inflammatory response, which is detrimental to the kidneys, because the risk of developing glomerulonephritis is increased.^[2,3,6–8]^ In return, chronic renal insufficiency promotes the onset or progression of PD.^[3]^ The involvement of chronic renal failure in the pathogenesis of PD is attributable to systemic inflammatory reaction, immunodeficiency, metabolic disorders, a weakened defense system against pathogens, and impaired self-repair capacity.^[4,11]^ A study has shown that 58.9% of patients with renal failure have moderate to severe PD.^[9]^ Mandalunis et al^[10]^ established a combined model of chronic renal failure and PD, and found that periodontal destruction was most severe in the combined model, thus suggesting interaction of the 2 diseases.

Efforts have been made to identify factors or causes that promote the progression of PD in ESRD patients. Evidence has shown that an increased proportion of Gram-negative bacteria in subgingival plaque flora in chronic renal failure patients may involve in the progression of PD.^[12]^ Moreover, a study of 115 hemodialysis patients revealed a positive association between the duration of hemodialysis and the severity of PD.^[13]^ In addition, PD is reported to be associated with malnutrition and inflammatory status, both of which are considered as predictors of morbidity and mortality in hemodialysis patients.^[9,14,15]^ Treatment decisions may be guided more appropriately if patients can be identified early as being at high risk of PD.

Although a clear relationship between chronic renal failure and PD has been reported, risk factors of developing PD in maintenance hemodialysis patients are still under investigation. In the present study, we compared the demographic and laboratory data of 136 uremic patients on maintenance hemodialysis to: analyze the prevalence of PD among hemodialysis patients of different ages; explore clinical characteristics of hemodialysis patients with PD as compared with their counterparts without periodontal disease; and identify the risk factors of PD in such patients.

## 2 Materials and methods

### 2.1 Subjects and grouping

From November 2015 to March 2016, uremic patients on maintenance hemodialysis at the Department of Nephrology, The First Hospital of Jilin University, were retrospectively reviewed. The inclusion criteria were: age ≥18 years and <80 years; glomerular filtration rate less than 10 mL/min/1.73 m^2^; at least 16 teeth in mouth, and at least 4 teeth can be evaluated (exclusion criteria were edentulous, cirrhosis, malignancies); subgingival scaling treatment within 1 month; receiving antibiotics and immunosuppressive therapy within 3 months; acute suppurative infection in oral cavity (such as tonsils or salivary glands) within 6 months; and periodontal treatment within 1 year. Eligible patients were divided into those with PDs (PD group) and those without PDs (non-PD group). This study was approved by the Ethics Committee of the First Hospital of Jilin University.

### 2.2 Periodontal examination

In this study, all dental examinations were performed by 1 dental specialist. The study followed the American Academy of Periodontology (AAP) guidelines.

The periodontal probing was performed with a modified pen-hold method. The probe was kept as parallel as possible to the long axis of the tooth. When close to the surface of tooth, the examiner moved the probe carefully until it encountered resistance of the bottom of sulcus, being careful not to probe the soft tissue or calculus at the bottom of the pocket. Then, the examiner probed the surface of each tooth to understand the position, range, depth, and shape of the pocket. Six loci were recorded for each tooth, including mesio, middle, and distal of labial (buccal) surface and lingual (palatal) surface. When the periodontal pocket of proximal surface was probed, the probe clung to the adjacent contact point of the tooth, and then the probe was tilted slightly toward the gingival col to detect the deepest part of the adjacent periodontal pocket. Periodontal probing was conducted in a certain order.

For measuring loose teeth, the anterior tooth was clipped with a dental forceps and shaken labiolingually; when examining the posterior teeth, with the forceps tip against the occlusal fossa, the examiner shook the posterior tooth buccolingually or mesial-distally.

### 2.3 Definition and diagnosis

Periodontal disease includes gingivitis and periodontitis. Gingivitis was diagnosed based on symptoms including red, swollen gums and prone to bleeding with mechanical stimulation, but without periodontal pocket or loss of periodontal attachment. Periodontitis was diagnosed on manifestations including gingival hemorrhage, formation of periodontal pocket, periodontal abscesses or periodontal abscess overflow, loose teeth, gingival recession, or atrophy. All patients were examined for PD by a professional dentist in our hospital. The oral examinations met the standards recommended by the World Health Organization (WHO), namely that at least 1 of 1/6 quadrant had not less than 2 functional teeth.^[16]^

### 2.4 Data collection

Demographic data—including age, sex, history of smoking, hypertension, diabetes mellitus, and frequency of teeth brushing each day—were recorded from each participant. Laboratory data were also collected, including peripheral white blood cell (WBC) count, hemoglobin, platelets, high-sensitivity C-reactive protein (hs-CRP), fasting blood glucose (FBG), total cholesterol, triglyceride, low-density lipoprotein-cholesterol (LDL-C), high-density lipoprotein-cholesterol (HDL-C), and serum levels of calcium and phosphorus and parathyroid hormone.

### 2.5 Statistical analyses

The SPSS for Windows version 19.0 software package (SPSS Inc., Chicago, IL) was used for the statistical data analysis. According to the formula 
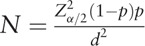
, *d* represents allowable error or admissible error (10% of prevalence), *p* the prevalence of PD in ESRD population (about 50%),^[9]^*α* = 0.05, and *Z*_α/2_ = 1.96. Thus, the sample size of this study should be at least 96 cases.

Quantitative variables were expressed as mean ± standard deviation (SD). Categorical variables were reported as numbers and percentages. Differences between groups were assessed using the *t* test. Data expressed in terms of proportions were compared using the chi-square test or Fisher exact test. Unconditional binary logistic regression analysis was applied to determine the risk factors of PD in maintenance hemodialysis patients. Odds ratios (ORs) and their 95% confidence interval (CI) were used to assess the independent contribution of each risk factor identified. *P* < .05 was considered statistically significant.

## 3 Results

**Figure 1:**
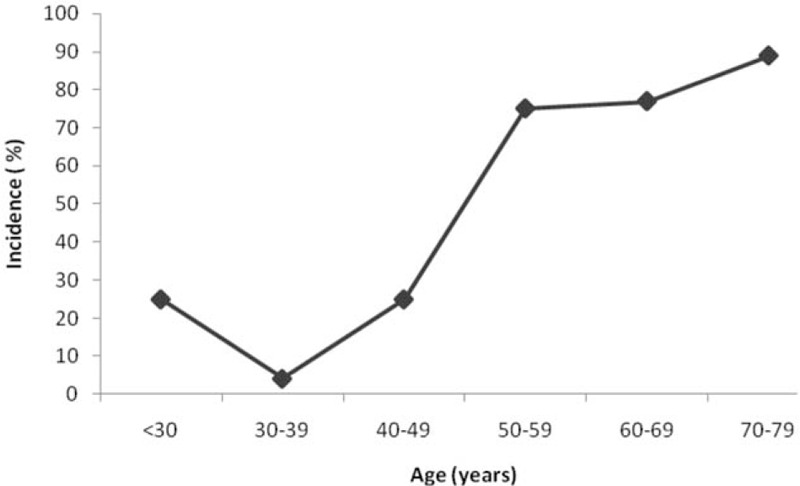
The incidence of periodontal disease among maintenance hemodialysis patients of different ages.

A total of 136 uremic patients (79 males and 7 females, aged 50.8 ± 15.3 years) on maintenance hemodialysis were included in this study. There were 70 patients (51.50%) in the PD group and 66 (48.50%) in the non-PD group. The etiology for ESRD included diabetic nephropathy (n = 49, 36%), chronic glomerulonephritis (n = 30, 22%), hypertension (n = 12, 9%), drug-induced kidney disease (n = 7, 5%), polycystic kidney disease (n = 4, 3%), and others (n = 34, 25%). Generally, 30 to 39-year-old hemodialysis patients had the lowest incidence of PD, and the incidence of PD increased with age, reaching 88.9% at the age of 70 to 79 years (Fig. 1).

Hemodialysis patients were most likely to develop PD if they were male, smokers, or diabetic (*P* = .009, <.001, and <.001, respectively). Patients brushing their teeth twice daily had significantly less chance of developing PD, as compared with those brushing only once daily (*P* < .001; Table 1).

**Table 1:**
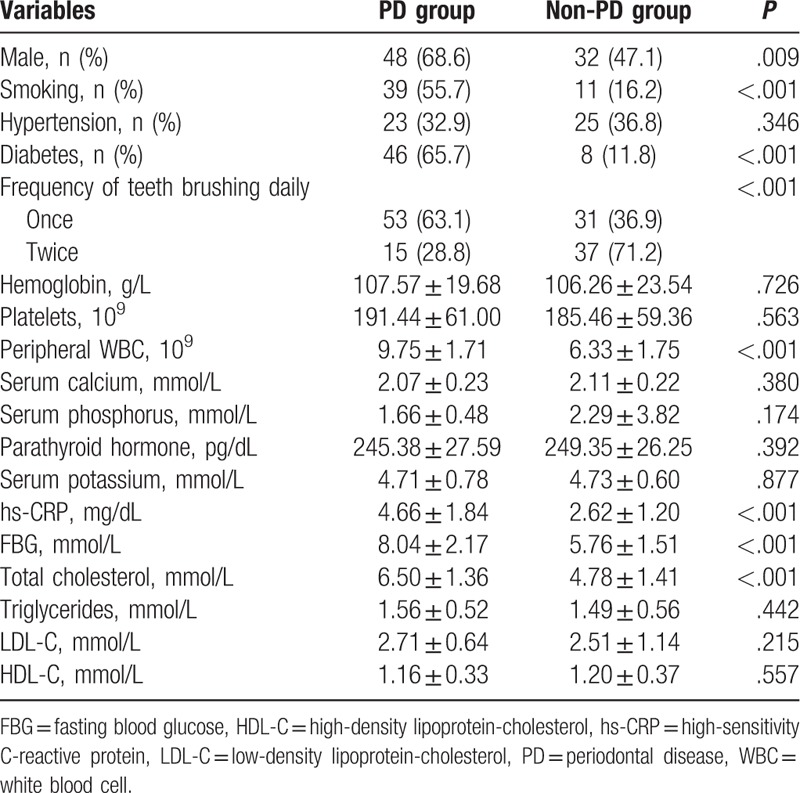
The characteristics of patients in the PD and non-PD groups.

Hemodialysis patients in the PD group had significantly high levels of total cholesterol, hs-CRP, FBG, and peripheral WBC count, compared with the non-PD group (all *P* < .001). There were no significant differences between the 2 groups regarding presence of hypertension, hemoglobin, platelets, serum calcium, serum phosphorus, parathyroid hormone, serum potassium, triglycerides, LDL-C, and HDL-C (*P* > .05; Table 2).

**Table 2:**
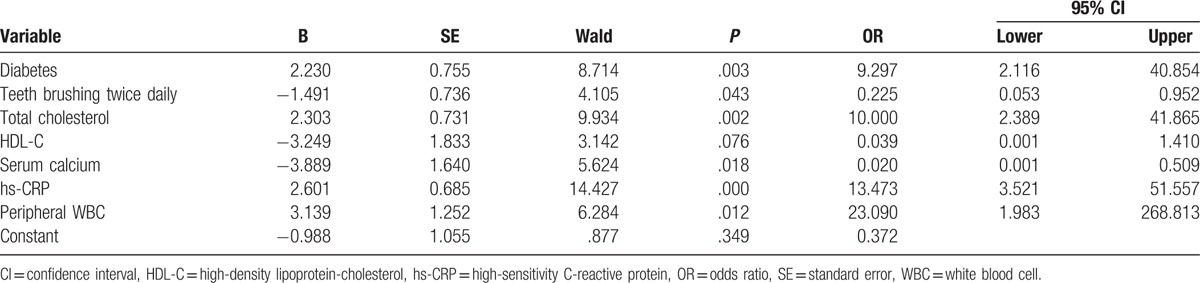
Risk factors of periodontal disease in maintenance hemodialysis by logistic regression analysis.

Logistic regression analysis revealed that diabetes (OR 9.297, 95% CI 2.116–40.854, *P* = .003), total cholesterol (OR 10.000, 95% CI 2.389–41.865, *P* = .002), hs-CRP (OR 13.473, 95% CI 3.521–51.557, *P* = .000), and peripheral WBC (OR 23.090, 95% CI 1.983–268.813, *P* = .012) were independent risk factors for developing PD among maintenance hemodialysis patients. Interestingly, twice-daily teeth brushing (OR 0.225, 95%CI 0.053–0.952, *P* = .043) and serum calcium (OR 0.020, 95% CI 0.001–0.509, *P* = .018) were favorable factors for maintenance hemodialysis patients against PD.

## 4 Discussion

Periodontal disease is characterized by loss of connective tissue attachment and alveolar bone destruction. Dental plaque microorganisms and their products are essential initiators of periodontal lesions. Previously, a close link between PD and uremia was reported.^[4,9,13]^ Identification of risk factors of PD in maintenance hemodialysis patients is conducive to early prevention of PD and improvement in quality of life among such patients.

Our data showed that 30 to 39-year-old hemodialysis patients had the lowest incidence of PD, and that the incidence of PD rose sharply with age reaching 88.9% at 70 to 79 years. Both physiological susceptibility and hygienic behaviors in the elderly contribute to a high incidence of PD. Given China's population has been aging rapidly in recent years, special efforts should be made in the early prevention and management of PD in the elderly population receiving hemodialysis.

Uremic patients typically have endocrinal disorders and metabolic abnormalities (even those on maintained hemodialysis), because regular renal replacement therapy is unable to completely substitute the function of kidneys. In this study, we explored clinical characteristics of periodontal status in hemodialysis, and identified the risk factors of PD in such patients. We found that uremic males had a higher chance of developing periodontal disease than females, which was consistent with the findings from the Third Chinese National Oral Health Epidemiological Survey. Also, a previous study revealed that longer hemodialysis was associated with severe PDs, especially in males.^[13]^ Furthermore, smoking seemed to increase the probability of PD, which partially explained a higher incidence of PD among males than females, because a higher proportion of males smoke. Smoking impairs the host response to microbial challenges on periodontal tissues, increases local calculus deposition, aggravates gingival inflammation, and enhances the destruction of the surrounding healthy periodontal tissues.^[17]^ As a preventive measure, twice-daily teeth brushing effectively prevented PD as compared with once-daily teeth brushing, suggesting the importance of sound hygienic behavior in the prevention of PD among hemodialysis patients.

Hemodialysis patients with diabetes or increased FBG are more likely to develop PD, and these results were further confirmed by logistic regression analysis, which revealed diabetes as an independent risk factor of PD. Current evidence suggests poorer glycemic control contributes to poorer periodontal health.^[18]^ Indeed, patients with diabetes are estimated to have 2 to 3 times greater risk of periodontitis.^[19]^ Two studies also showed that glycemic control worsens in parallel with the worsening of PD.^[20,21]^ In fact, periodontal inflammation has also been reported to exacerbate diabetes and worsen hyperglycemia.^[22]^ Recognition of the bilateral relationships between diabetes and PD will facilitate the better management of those patients’ concomitant diabetes and PD.

Whether hyperlipidemia is a contributor to PD still remains controversial.^[23,24]^ A previous study revealed that despite of a direct correlation between periodontal status and increased total cholesterol/triglyceride levels, none of these correlations turned out to be significant.^[25]^ Another study also supports no significant relationship between PD (regardless of its intensity) and blood lipid levels.^[26]^ However, another study indicated an increased ratio of total cholesterol to HDL-C was associated with PD. Moreover, serum proinflammatory cytokines, including tumor necrosis factor (TNF)-α and interleukin (IL)-1β, may contribute to the association between PD and hyperlipidemia.^[27]^ Similarly, we found increased total cholesterol level (as an independent predictor for the occurrence of PD) indicated the significance of cholesterol-lowering therapy for improvement of periodontal status in hemodialysis patients.

In this study, increased peripheral WBC count and hs-CRP were found to be associated with PD in hemodialysis patients. Periodontitis is an inflammatory disease initiated by oral microbial inflammation, which activates the innate immune system resulting in the release of proinflammatory cytokines and recruitment of phagocytes and lymphocytes.^[23,28]^ This inflammatory scenario and toxins further drive the destruction of periodontal connective tissue and alveolar bone. The interaction of these complex factors caused a systematic disorder, including periodontal lesions, system inflammation, and uremic toxins, which further interacted in a vicious cycle.

This study showed that there were no significant differences in serum calcium and phosphorus levels between the PD and non-PD groups. The probable reason may be that all patients in this study were uremic patients on maintenance hemodialysis presenting with typical abnormities in mineral metabolism, manifested by high phosphate, low calcium, and secondary increase in parathyroid hormone secretion. However, the logistic regression analysis revealed serum calcium as a protective factor for maintenance hemodialysis patients against PD. A previous study consistently found that higher calcium intake might be associated with decreased prevalence of PD.^[24]^ Thus, supplementation with calcium seemed to be favorable for preventing PD among hemodialysis patients.

This study has several limitations. Firstly, it is a single-center, retrospective cohort study with a relatively small sample size, which results in unavoidable selection bias. Secondly, the extent to which lifestyle, dietary habits, and pharmacological interventions affected our study population is not known. Thirdly, the study did not include health controls, which limited the analysis for comparing the risks of PD in hemodialysis and healthy patients. Fourthly, the role of some unmeasured confounding factors that could have possibly influenced the observed association cannot be entirely ruled out. Finally, the patients were divided into a PD group and a non-PD group, without further distinguishing gingivitis, acute periodontitis, chronic periodontitis, or invasive periodontitis. These limitations could be overcome by a larger-scale, prospective longitudinal study in the future. Considering this is a preliminary observational study, mechanistic studies are required to elucidate the underlying correlation between those variables and PD in hemodialysis patients.

## 5 Conclusions

In conclusion, the incidence of PD increased with increased age among uremic patients on maintenance hemodialysis. Diabetes, total cholesterol, hs-CRP, and peripheral WBC were independent risk factors for developing periodontal disease, whereas twice-daily teeth brushing and serum calcium were favorable factors for maintenance hemodialysis patients against PD. Knowledge of these pathological changes provides a theoretical basis for the prevention and improvement of PD, such as smoking cessation, cultivation of sound hygienic behavior, glycemic control, lipid modification, correction of inflammatory reactions, and supplementation with calcium.
